# Strategies to strengthen non-governmental organizations' participation in the Iranian health system

**DOI:** 10.3389/fpubh.2022.929614

**Published:** 2022-11-28

**Authors:** Arman Sanadgol, Leila Doshmangir, Reza Majdzadeh, Vladimir Sergeevich Gordeev

**Affiliations:** ^1^Student Research Committee, Department of Health Policy and Management, School of Management and Medical Informatics, Tabriz University of Medical Sciences, Tabriz, Iran; ^2^Social Determinants of Health Research Center, Tabriz University of Medical Sciences, Tabriz, Iran; ^3^Department of Health Policy and Management, Tabriz Health Services Management Research Center, School of Management and Medical Informatics, Tabriz University of Medical Sciences, Tabriz, Iran; ^4^Interdisciplinary Research and Practice Division, School of Health and Social Care University of Essex, Colchester, United Kingdom; ^5^Institute of Population Health Sciences, Queen Mary University of London, London, United Kingdom; ^6^Department of Infectious Disease Epidemiology, London School of Hygiene and Tropical Medicine, London, United Kingdom

**Keywords:** non-governmental organizations, NGO, health system research, health policy, health system, health system strengthening

## Abstract

**Background:**

Non-governmental organizations (NGOs) added a new dimension to intersectoral action for health. Involving the NGOs in health system strengthening could lead to a more efficient, equitable, and better-governed healthcare system. This qualitative study explored effective strategies for NGO participation in the Iranian health system to achieve broader health system goals.

**Method:**

We conducted 33 semi-structured interviews with health policymakers and planners, NGO actors at the national and provincial levels, and other key informants. The qualitative data were analyzed through a thematic analysis approach. Trustworthiness in the study was observed at all stages of the study.

**Result:**

Four main themes- were identified: empowerment for learning leadership and management skills, creating active participation in policy-making, capacity building for participation, clarifying participation process, falling into 17 sub-themes. Along with the government and health sector policymakers, NGOs may have a significant role in improving health system goals and increasing equity, social responsiveness, financial risk protection, and efficiency.

**Conclusion:**

The participation of NGOs in the Iranian health system is a complex process. All elements and dimensions of this process need to be considered when developing a platform for the appropriate participation of NGOs in the health system functions. Evidence-informed strategies for strengthening the participation of NGOs in the health system should be used to utilize NGOs potential to the fullest.

## Introduction

To promote fairness in the health system and facilitate cross-sectoral collaboration, it is necessary to involve all actors to achieve better health for all (1). The greater the participation, the more favorable are health system outcomes, access and equity (2, 3). Participating stakeholders can include formal organizations with economic interests andpeople and community-based organizations (4). It is also critical to understand the role the various groups and organizations participation can play in health system functioning (5). In recent years, utilizing inter-sectoral approaches has been a critical strategy for health systems (6). Collaborations and partnerships can bring certain benefits, including creating new opportunities, mobilizing resources, addressing health needs, increasing access to health care and health services, reducing health costs, increasing the quality of services provided, and increasing the population covered (7–10).

In an attempt to achieve the health system goals, governments cannot act independently without relying on the capacities of society, community participation and their capacity, and they need to turn their attention to various groups, including non-governmental organizations (NGOs) (11, 12). Therefore, it is time to consider the emerging role of NGOs are non-traditional players that should be engaged in health service provision, as they are more flexible and innovative. They can also help achieve goals and lead to safe, effective, efficient and equitable health services (13–16).

NGOs can provide needed health services and supplement or, in some instances, extend government services (17, 18). Failure to integrate NGOs in the health care service provision process and weak inter-sectoral collaboration can hinder overall progress toward improving health performance (19). Involving the NGOs in health system strengthening could lead to a more efficient, equitable, and better-governed healthcare system and better health system performance (20–22). However, the engagement of NGOs in health service provision is not straightforward, as they face specific challenges in different areas, including financing, financial management, human resource management, resource mobilization, networking, government support, access to information, and strategic planning (23–26). To overcome the listed challenges, NGOs' participation strategies need to be explored further and understood better.

### Context

According to unofficial statistics, in Iran, in 2020, there were about 20,000 NGOs. The first legal document on the participation of NGOs in the Iranian health system dates back to 1980 (27). The role of NGOs in public health and health care since the beginning of their establishment in Iran has been health care provision, treatment and pharmaceutical activities, financial support, education and prevention, social health, consulting role and technical information, research, information activities, facilitation activities, advocacy, as well as the role of monitoring of individuals' rights in the community (27–29). NGOs have existed in Iran for a long time. They are funded by various donations, including donations by citizens and other charitable and government donations. They provide various health care services across different medical fields, including infectious diseases, endocrine and metabolic diseases, blood and genetic diseases, substance and alcohol and smoking abuse, cancer, neurology and mental health, social health, blindness and deafness, kidney and liver disease, disability and injury, heart disease and maternal health care and pregnancy (28, 30–35). In Iran, similar to other countries, there were particular successes and failures for various reasons. Effective collaboration could be essential to achieving the full potential. Hence, we use the Iranian setting to explore which strategies from stakeholders' perspectives could work and which will not. The findings of this study can assist policymakers and planners in resolving the NGOs' participation challenges and enhancing the effectiveness of their collaboration. This study aimed to explore the views of NGOs' participants, policymakers and planners NGOs regarding the use of different strategies to strengthen the participation of NGOs in Iran's health system.

## Materials and methods

### Design and setting

This qualitative case study was carried out using semi-structured interviews and documentary reviews. The Consolidated Criteria for Reporting Qualitative Research (COREQ) 32-item checklist was followed for reporting qualitative data (36) (Appendix 1). All participants provided written informed consent before participating in interviews. The research team consisted of four academic researchers, three males, and one female. All four research team members had prior experience in qualitative research.

### Participants

Semi-structured interviews were conducted with NGO managers, board of directors and technical experts, faculty members, researchers, scholars, heads of NGOs and experts of government agencies that issue licenses to NGOs' establishments. Considering the scope and role of NGOs and the geographical extent of Iran, purposeful sampling with maximum variation was used to select participants. In addition to practice backgrounds and experience, for NGOs, criteria such as the level of activity (provincial, national, and international), the number of years of activity, and their willingness to participate were considered. For the rest of the participants, individuals' educational and professional backgrounds were also considered when selecting interviewees. People with experience in collaboration with health organizations and involvement in related activities were preferred. Before participation, the prospective participants were contacted and invited to participate in the study *via* email or telephone, and the interview was conducted face to face. Data saturation were considered as the basis for the number of study participants. Interviewee characteristics are presented in Appendix 2.

### Interview tool and data collection

We developed a semi-structured interview guide that comprised five parts—participants' demographic data, current professional roles, study objectives, informed consent, and general opinion (main study questions). The interview guide was validated by three study participants and trialed under intended interview conditions. With participants' consent, all interviews were audio-recorded, and each lasted about 38 min. An independent interviewer conducted all interviews. A total of 33 semi-structured interviews took place.

### Data analysis and trustworthiness

All interviews were transcribed verbatim. Analyses were performed simultaneously with transcription. If there were any inconsistencies or ambiguities, they were resolved by calling back participants for clarification. In order to ensure the data were robust, strategies of member checking (in three stages of data collection, at the end of each interview and after data analysis) and auditing (after extracting initial codes and development of initial themes) were used. All interviews content was analyzed using the thematic analysis assisted by MAXQDA 12 (VERBI Software. MAXQDA *12*. Berlin: VERBI Software, 2015). Data coding process was done in 3 steps; generating and finalizing the initial codes, grouping codes denoting the same concepts into subcategories, classifying subcategories indicating similar subjects into the main categories. Two authors read all documents and transcripts of the interviews to extract codes, subthemes and themes. During the data analysis phase, the two researchers had regular meetings to discuss any disagreements openly to reach a consensus. When consensus was not reached, the other two researchers were involved in resolving any disagreements.

## Result

We structure our findings based on results from the thematic analysis of interviews, where we identified four main themes and 17 sub-themes (Table 1).

**Table 1:** Theme and sub-themes of strategies to strengthen NGO participation.

**Theme**	**Sub-theme**
Empowerment for leadership and management skills	Communication skills Management and decision-making skills Knowledge of public health
Creating active participation in policy-making	Building culture Political will and commitment A legal framework for participation Transparency in health system decisions Policy alignment with NGOs' targets
Capacity building for participation	Identification of participation barriers Individual and institutional competencies for participation Legislations authority for participation in policy design and implementation Relationship between government and NGOs
Clarifying participation process	Transparency of the participation process Access to information and knowledge Familiarity with a variety of conversation techniques Existence of stable financial resources Long process of participation arrangement

### Empowerment for learning leadership and management skills

Because NGOs are more concerned with the target community with specific problems, communication skills are very important and need to improve skills such as active listening, friendly communication based on respect, empathy, and effective response to their target community. participants also stated, as NGOs constantly cooperate with the government and various departments, domestic and international NGOs, all NGO employees should be trained and improve their communication skills.

“*Unfortunately, active members of NGOs suffer from a lack of communication skills in relation to various individuals and organizations”. [A manager at NGO]*

Several respondents had the opinion that working in NGOs is a multidimensional activity. They argued, for example, that in order to attract funds, NGO members should be familiar with financial knowledge and marketing and sales techniques to promote themselves in the region. Therefore, they should learn about the health system's functions and goals, such as service delivery and resource generation.

“*Today, an NGO is like a private organization that should know about financial management, marketing and sales departments*”. [*A researcher in NGOs*]

In addition, some other respondents referred to managerial skills and specialized knowledge. They suggested that these skills should be developed with the help of the government or the Ministry of Health and Medical Education (MoHME) to enable NGOs in assisting the government.

“*As long as NGOs do not move toward learning specialized skills and knowledge, they will have difficulty in making decisions and prioritizing”. [A senior health official]*

NGOs work to protect, promote and restore the health of people in the community, and in this regard, the participants stated that it is necessary to use public health knowledge and NGOs should be empowered in this regard.

“*NGOs must have the necessary and sufficient knowledge and skills in the field of public health, and if they have a lack of knowledge, the government and health authorities must train them”. [A senior health official]*

### Creating active participation in policy-making

Respondents stated that the participation of NGOs in health policy-making has improved in recent years. However, they also mentioned that NGOs still do not have a significant role in the health system and are not adequately engaged in the policy-making process.

“*Unfortunately, although NGOs can play an important role in helping the government, they are ignored”. [A manager at NGO]*

Some of the respondents mentioned the existence of a culture of participation as one of the most critical factors. They stated that until the issue of participation in decision-making becomes a principle and becomes a culture, the capacity of NGOs in health policymaking will be limited.

“*I think it is essential to have a space for NGOs' participation in health system programs”. [A board of directors of NGO]*

Others cited decision-makers' political will and commitment as an issue for NGOs' participation. They explained, for example, that the change of government should not change the status of NGOs. It would also be necessary to accept NGOs' existence and activities as a fundamental principle. Since NGOs' capacity is being utilized continuously, decisions related to their work should be more transparent.

“*As long as governments and health policymakers are not interested in NGO participation, NGO efforts will not be well visible”*. [*A NGOs researcher*]

For many respondents, a very important issue regarding the activities of NGOs in Iran is the lack of proper legal documents and support for them, while existing laws are not fully enforceable and it seems that this issue should be one of the priorities of the government and a legal path that can facilitate their activities should be on the agenda. So, one of the necessary tools is the existence of applicable laws and frameworks.

“*I think the existing laws have done little to help NGOs, and in many cases are even deterrents”. [A NGO' staff]*

Many NGO participants stated that the Iran's health system is not transparent and that they do not have a proper understanding of activities, measures and goals, and that with the change of government, these things will change.

“*Too often, decisions are not transparent or do not give us feedback on our impact on the programs we do”. [A manager at NGO]*

Iran‘s health policies can be divided into two parts, stable policies and policies that change over time and the problems that arise, such as the during coronavirus. For this reason, participants stated that the activities of NGOs can vary under the influence of donors and their organizational goals, but ultimately these goals and policies must be in line with government goals, which requires closer proximity and more oversight of NGOs.

“*The programs and mission of NGOs, both domestic and foreign, must be in line with the policies of the Ministry of Health and be in the same direction”. [A senior health official]*

### Capacity building for participation

Almost all participants believed that have many problems and barriers in both the government and NGO structure that prevent them from effectively addressing health problems, and the government should strive to identify and address them.

“*NGOs will not be able to run at full capacity until existing issues are resolved”. [A board of directors of NGO]*

Most respondents emphasized that there is little space for participation, and the government should develop strategies and a straightforward process for greater NGO participation and support. They also underlined existing gaps in the legislature.

“*In the country, the capacity of NGOs should be used in decision-making, policy-making and implementation of programs”. [A researcher in NGOs]*

NGOs should also empower themselves and improve their capabilities to achieve effective performance in aiding the government's goals. They should also be given the advisory role, given their active presence in society and familiarity with the existing health problems, especially among vulnerable groups.

“*One of the most important capabilities of NGOs is their involvement in judicial health affairs to be the voice of the people”. [A NGO' staff], “NGOs can be used as advisors in policy and decision-making meetings”. [A manager at NGO]*

### Clarifying participation process

Participants stated that the government and NGOs should engage in long-term planning and partnership to make collaboration sustainable. They explained that the overall participation process should be clearly defined, and considerable attention and support should be given to sustainable time, financial and human resources.

“*We have to accept that any change in the NGO's participation process is long-term, and that change in the short term is often detrimental”. [A researcher in NGO], “One of the primary measures that the government must consider for NGOs is having sustainable financial resources”. [A researcher in NGO]*

Respondents also stated that the issue of NGOs' access to specialized knowledge in their field of activity is critical. This knowledge should be up-to-date to use various policy-making techniques, such as policy dialogue, whenever necessary.

“*In my opinion, given the free access to information in the world and the sanctions in Iran, the government should take measures to ensure that NGOs have access to information so that they can use their capacity”. [A manager at NGO]*

Based on respondents' feedback, the government should have a fundamental responsibility and commitment to engage with NGOs and other partners and sectors within the health sector. It should also focus more on the dynamic interrelationships between capacity strengthening and the process of NGO participation in the health sector. Finally, the government should allocate budgets and develop policies for interaction, training, and empowerment of these organizations working in the health sector. Respondents stated that no matter how hard the health systems and health professionals work toward the goals of the health system, these goals will not be achieved unless the health systems and all stakeholders are ready to engage and participate together and are supported by the government that designs and deploys appropriate and effective strategies in this regard.

“*In order to participate, the tasks and roles of NGOs must be clear”. [A manager at NGO], “In my opinion, given that one of the requirements for NGOs to show themselves in the policy-making process is their negotiating power and familiarity with negotiation techniques”. [A senior health official]*

## Discussion

We identified empowerment for leadership and management skills, creating active participation in policy-making, capacity building for participation and clarifying the participation process are essential to strengthen the participation of NGOs in the health system.

Gaps and flaws in countries' health systems prevent them from achieving their goals effectively and efficiently, and in fact, NGOs can fill these gaps. Government partnership with NGOs to strengthen the health system can lead to increased equity and efficiency, and NGOs' engagement with the public sector is seen as instrumental in attending to the issues of equity and quality improvement of the services provided along with dealing with the issues of access and responsiveness of the system (21). Since the NGOs are more flexible than the public health sector, they can attract more people's attention to specific diseases such as HIV/AIDS, leading to more people being covered in the health system (37). Gross improvements in the quality and efficacy of medical care would require strengthening the government's health programs and would surely necessitate collaboration with NGOs (38).

Our study found empowerment among the themes for strategies participation in a strengthening non-governmental organizations in the health system. The concept of “empowerment” started to become more popular after the Beijing Fourth World Conference in 1995. The Conference documents had declared empowerment as a prerequisite to achieving health for all people (39). Community participation is another essential part of the process of good local governance, and empowerment remains at the heart of effective health promotion. Community participation and empowerment are two core principles underpinning the Healthy Cities movement (40). Other studies also cite the empowerment of NGOs as a necessary precondition for promoting community health (41, 42). NGOs can reduce government financial costs and provide the necessary workforce. They are paving the way for a proper flow in moving toward the health system's goals (43, 44).

In the health system, NGOs can provide various services from prevention to treatment to their target community to strengthen the health system and increase equity and access to health services (22, 45–53). In order to use NGOs effectively and efficiently, governments must plan national health system strategies based on the appropriate policies of their own country. They should also provide NGOs with standards for care, ensure equity in service delivery, harmonize information systems, achieve geographic coverage, and carry out long-term planning based on local health priorities (54). There is a need to change the beliefs system and attitudes in providing services in the country's health system, and the capacity of NGOs should be used more (55).

Some countries have a set of comprehensive laws to support NGOs' participation in the policy-making process. However, Iran continues to suffer from the lack of a comprehensive law for NGOs' activities. Moreover, there are no specific structures to engage NGOs in policy-making (56), which is among the most critical barriers (31). Nonetheless, studies conducted in health in Iran have always considered NGOs a stakeholder (e.g., hepatitis C, HIV/AIDS, physical rehabilitation services (57–59).

There are numerous examples of government partnerships with NGOs that demonstrate increased equity in healthcare access. For example, in Malawi, the public sector, with NGOs' help, has delivered treatment to a large number of patients relatively quickly with promising outcomes (60). In East Timor, NGOs strengthened the country's health system in the post-crisis period (61). In Ethiopia, the activities of NGOs opened the space for the development of health services (62), and in Kenya, NGOs also reduced out-of-pocket payments for health care (57).

In order to influence the health system, NGOs must have the necessary management training such as decision-making, organizational vision, mission and strategy to perform well (58, 59, 63, 64). NGOs also need sustainable funding and management training. NGOs must also work continuously with different parts of the health system in a country (65). In addition to the political will, adequate funding is crucial to strengthening the health system and working with NGOs (66). The capacity of NGOs can also be used in health policy-making, whether as an advisor, implementers or monitors health service quality (28, 67, 68). However, governments can use the capacity of NGOs to strengthen their country's health system, which requires long-term planning, adequate funding, and a culture of participation (28). Policy barriers need to be removed by creating a necessary legal framework, developing trust and societal acceptance of NGOs' activities and involvement, and training and empowering NGOs in health (3, 31, 32, 69). Also, the latest research on the role of NGOs in Iran's health system found that NGOs play different roles in Iran's health system, but they are ignored and appropriate strategies need to be applied (29).

### Study limitations

Study participants had different meanings from the terms such as NGO participation in health system, trust to government and the actual role of NGOs and each person explained these terms from their own perspective. And also some of the participants were not familiar with the health system goals and its function. Due to nature of the qualitative study, generalizability was not the researchers' aim.

### Policy implications for the policy-makers

The results of our study showed that the participation of NGOs can play a major role in strengthening the health system, but the important point is that health policy makers and planners define a plan and strategies on how to involve them, as well as policymakers using evidence based tools to design effective strategies.

## Conclusion

The government needs significant policy interventions in the health system to strengthen the role of NGOs. Along with the government and other health sectors, NGOs can play a crucial role in improving health system performance and increasing equity, responsiveness, social and financial risk protection and efficiency. The participation of NGOs in the goals of each country's health system requires the creation of new ideas of cooperation and their acceptance and should try to remove barriers to the participation of these organizations in health system functions and goals. Strengthening NGOs is a shortcut to achieving the health system's goals, which can be achieved *via* qualitative and quantitive strengthening of health activities in NGOs and specialization of their activities, as well as building trust of decision-making bodies in the work of NGOs. In this regard, the governments of each country should use appropriate strategies to use the capacity of these organizations.

## Data availability statement

The original contributions presented in the study are included in the article/Supplementary material, further inquiries can be directed to the corresponding author/s.

## Ethics statement

This study was approved by the Ethics Committee of Tabriz University of Medical Sciences (Approval No: IR.TBZMED.REC.1399.370). The patients/participants provided their written informed consent to participate in this study.

## Author contributions

LD conceived the study. AS and LD contributed to designing, collecting, analyzing, drafting, and finalizing the paper. RM and VG contributed to analyzing the data. All authors approved the final version of the paper.

## Funding

The study was funded by Tabriz University of Medical Sciences, Tabriz, Iran (Grant No: 64241).

## Conflict of interest

The authors declare that the research was conducted in the absence of any commercial or financial relationships that could be construed as a potential conflict of interest.

## Publisher's note

All claims expressed in this article are solely those of the authors and do not necessarily represent those of their affiliated organizations, or those of the publisher, the editors and the reviewers. Any product that may be evaluated in this article, or claim that may be made by its manufacturer, is not guaranteed or endorsed by the publisher.

## Abbreviations

NGOs: non-governmental organizations.
