# Supplementary material for: Strategies to strengthen non-governmental organizations' participation in the Iranian health system
Source: Front Public Health. 2022 Nov 28;10:929614. doi: 10.3389/fpubh.2022.929614 (PMC9742430; doi:10.3389/fpubh.2022.929614)
Supplement: Supplementary file 2 [file Table_2.DOCX]

**SUPPLEMENTARY MATERIAL**

**Strategies to strengthen** **non-governmental organizations’ participation in the Iranian health system**

**Appendix 2:** Baseline characteristics of study participants

| Participant's characteristics | Number (%) |
| --- | --- |
| **Age (year)** | |
| ≤ 30 | 5 (15) |
| 31-40 | 17 (52) |
| 41-50 | 7 (21) |
| >50 | 4 (12) |
| **Gender** | |
| Male | 14 (42) |
| Female | 19 (58) |
| **Education** | |
| ≤ Diploma | 3 (9) |
| BSc^a^ | 8 (24) |
| ≥ MSc^b^  Notes: ^a^ Bachelor of science;  ^b^ Master of science | 22 (67) |
| **Job position** | |
| Manager/board of director of NGO* staff | 19 (58) |
| Head of unit | 2 (6) |
| Expert of unit | 4 (12) |
| Researcher and faculty member | 8 (24) |
| **Work experience (year)** | |
| 5-10 | 12 (36) |
| 11-15 | 11 (33) |
| >15 | 10 (31) |
| **NGOs activities (*among NGO staff)** | |
| Cancer | 2 (10.7) |
| Phenylketonuria | 1 (5.2) |
| Autism | 1 (5.2) |
| Epilepsy | 1 (5.2) |
| Hemophilia | 1 (5.2) |
| HIV/AIDS | 2 (10.7) |
| Diabetes | 1 (5.2) |
| Thalassemia | 1 (5.2) |
| Multiple sclerosis (MS) | 1 (5.2) |
| Specific diseases | 2 (10.7) |
| Kidney | 1 (5.2) |
| Mental diseases | 2 (10.7) |
| Social health | 1 (5.2) |
| Development of public health | 1 (5.2) |
| Reduce social harms and diseases | 1 (5.2) |
